# Orbital floor fractures – short- and intermediate-term complications depending on treatment procedures

**DOI:** 10.1186/s13005-015-0096-3

**Published:** 2016-01-05

**Authors:** Henrik Holtmann, Hatice Eren, Karoline Sander, Norbert R. Kübler, Jörg Handschel

**Affiliations:** Department of Oral and Maxillofacial Surgery, Plastic Surgery of the Face, University Hospital of Duesseldorf, Moorenstr. 5, D-40225 Duesseldorf, Germany; Department for Oral Surgery, University Hospital of Duesseldorf, Moorenstr. 5, D-40225 Duesseldorf, Germany

**Keywords:** Orbital floor fracture, Surgical reconstruction, Titanium mesh, Resorbable polydioxanone foil, Internal orbital reconstruction

## Abstract

**Background:**

Many reconstruction materials for orbital floor fractures have been described in the past including autologous bone transplants, resorbable polymers and titan meshes. So far evidence is missing which material is used successfully regarding indication and particular size of defect. Therefore the aim of this study was to evaluate which reconstruction technique produces best clinical outcome and least complications associated with indication.

**Methods:**

Retrospectively, surgical and ophthalmological data plus CT scans from a collective of 775 patients between 2005 and 2012 were analyzed. Furthermore included patients were sounded on satisfaction and potential problems postoperatively.

**Results:**

Overall 593 patients offered full pre- and postoperative short-time data appropriate to inclusion criteria – of these 507 (85,5 %) underwent primary surgical treatment. Smallest average defect size was found in cases with no indication for surgical treatment (81 mm^2^), largest in cases indicating titanium mesh reconstruction (601.5 mm^2^). In 15 cases exact fragment reposition was possible without insertion of alloplastic material. Best clinical results obtained reconstruction using polydioxanone foil (PDS). 0.15 mm PDS-foil: 444 patients, reduced diplopia pre to postoperative 16 to 6 % (*p* < 0.01), ex- and enophthalmus < 2 % after surgery. 0.25 mm PDS-foil: 26 patients, reduced diplopia from pre- to postoperative 34,6 to 3,8 % (*p* < 0.01), postoperative exophthalmus rate was higher than preoperative (3,8 to 7,7 %). In comparison to reconstruction with PDS-foil a higher percentage of patients reconstructed with titanium meshes (*n* = 22) revealed no significant reduction of diplopia (45,5 to 31,8 %; *p* = 0.07). Furthermore 63 of all included patients agreed to complete a questionnaire on intermediate-term postoperative symptoms and surgical contentedness. Remarkably 50 % of the patients reconstructed with titanium meshes indicated foreign body sensations and cold feeling in the long-term.

**Conclusions:**

Short- and intermediate-term results of clinical outcome in our patients with surgical treated orbital floor fractures (i.e. diplopia, en- or exophthalmus) reveal that thin resorbable foils, particularly 0.15 mm diameter PDS-foil seem to generate best results referring to orbital floor defects with a size of 250 to 300 mm^2^.

**Trial registration:**

Study number 4222, year 2013, ethics committee of the medical faculty of the Heinrich Heine university of Duesseldorf.

## Background

In the past years a lot of reconstruction principles and materials have been described for orbital floor fractures. Regarding the management of orbital floor fractures there is still no profound reliable consensus regarding the indication for surgery, best time of surgery and the risk for late enopthalmus. Often the decision for surgery is based on individual and local traditions all over the world [1] and first treatment algorithms are established [2] but not proceeded in the whole world.

Concerning the materials that are used to reconstruct the fractured orbital floor the trend is leaving autogenous (bone) grafts to well tolerate alloplastic materials emerged in the past few years. But even in the field of reconstruction materials there is no reliable consensus which material is the best to use. Often the decision for a certain material depends on surgeon preference, experience and comfort [3].

Mok et al. [4] performed a review of the literature that gained to find out the optimal material for reconstruction defining the following required characteristics: resorbable, osteoconductive, resistant to infection, minimal reactive, no capsule formation, half-life which would allow significant bony ingrowth, cheap and readily available. Summarizing these requirements, the article concludes that no material that is actually being used unites all these attributes. Therefore Avashia et al. developed a decision-making algorithm for orbital floor reconstruction materials in 2012 by reviewing all available literature [5]. Even in this retrospective analysis the authors bewail a lot of flaws in the study designs of the included literature so that no closing statement can be given which material should be used preferrably for orbital floor fracture reconstruction. Strong EB et al. confirm this statement in a current study [6]. Leaving these definition attempts actual published literature shows that especially alloplastic materials are mainly used to reconstruct the destroyed orbital floor due to their current availability, clinical and operative cost and their overall ability for well-tolerance [3].

One of these important alloplastic materials is titanium. In 2003 Ellis 3^rd^ and Tan were able to show that titanium meshes are more accurate to reconstruct the orbital floor than bone grafts [7]. Kirby et al. confirmed these findings in a retrospective study with 317 adult patients comparing patients who had been reconstructed with bone grafts to patients reconstructed with titan-mesh and/or polyethylene [8]. Even though chronic enophthalmus (persisting more than 4 weeks) was significantly reduced in those patients with alloplastic reconstruction, (23 to 14 %) diplopia was increased (17 to 14 %) in comparison to patients reconstructed with bone grafts. A new publication with 144 patients reconstructed with preformed titanium-meshes showed low postoperative rates of enophthalmus (3,8 %) and diplopia (2,2 %) [9].

In 2004 Ellis 3^rd^ and Messo additionally estimated that titanium meshes and porous polyethylene are more compatible to reconstruct orbital floor fractures than for example silicone or Teflon® and even better than resorbable alloplastic materials like polydioxanone (PDS) membranes [10]. Polydioxanone maintains up to 12 month in the orbital soft tissue until full degradation, sometimes degradation seems to be incomplete and granulomas occur more often due to a sterile inflammatory cellular reaction lining the degrading polymer fragments. On the other hand a vigorous fibrotic reaction can also be seen in patients with titanium mesh implants [11]. And furthermore an increased rate of infection, extrusion, implant migration and residual diplopia has also been reported for patients reconstructed with titanium meshes [12]. Kontio et al. used pre- and postoperative CT- and MRI-scans which showed unsatisfactory reconstructed orbital shapes after reconstruction with polydioxanone membrane (thickness 0.25 mm or 1 mm), revealing not adequately restored orbital volume and thick soft tissue scar formation [13]. Unfortunately this study only consisted of 16 patients and defect size was not mentioned. Baumann et al. also examined polydioxanone (0.25 or 0.5 mm thick) reconstructed orbital floor fractures and concluded that polydioxanone should only be used in fractures up to 2.5 cm^2^. Patients with larger defects had an increased risk of developing enophthalmus due to its missing stiffness and stability to hold back the orbital soft tissue. Evaluationg radiographs they also discovered that newly build bone did not occur in the defects after resorption of polydioxanone [14].

Gierloff et al. extended the indication for using polydioxanone foils. Inter alia 47 patients with defects > 2.0 cm^2^ were treated using foils with a thickness of 0.25 mm and revealing a reduction preoperative to 6 month postoperative of eye mobility disorders from 31 to 5 % and enophthalmus from 5 to 2 % [15]. Gerressen et al. reinforced these findings in 21 patients evaluating postoperative CT-scans. They described the usefulness of polydioxanone foil (0.25 mm) or ethisorb patch reconstructing the orbital geometry even in cases with extensive fractures (median: 4.32 cm^2^) comparing the results to the healthy side [16]. A significant reduction of diplopia from 57 % (preoperative) to 19 % postoperative (median: 27.4 month postoperative) was shown.

A current study enrolling 78 patients could even show no relevant functional difference using neither resorbable nor titanium-dynamic mesh plates concerning defect size and reduction of enophthalmus, extraocular movement disorders and diplopia [17].

Although a lot of approaches were made to define whether titanium meshes are more effective than absorbable materials to reconstruct the fractured orbital floor (particularly polydioxanone sheets) as seen above this question remains unacknowledged up to now due to fact that no material has only advantages. Even the question on which material is best choice for which size of defect cannot be answered properly regarding the present international literature so far. Therefore the purpose of this study is to evaluate alloplastic materials (polydioxanone foil with a thickness of 0.15 mm or 0.25 mm and titanium meshes) currently used in a large number of patients specifying which material should be used for which indication in purpose of a successful reconstruction.

## Methods

### Study design and methods

We performed a retrospective study for a collective of 775 patients with orbital floor fractures (isolated or combined to other midfacial fractures) admitted to and treated in the clinic of oral, maxillofacial and plastic facial surgery at the university of Duesseldorf, Germany in the years 2005–2012.

Data basis was all clinical data of the patients including preoperative CT-scans, preoperative ophthalmological consultations and ophthalmological consultations 2 weeks postoperative. Target parameters and data inclusion criteria were pre- and postoperative diplopia, extraocular movement disorders, exophthalmus, enophthalmus and primary surgical treatment.

**Fig. 1:**
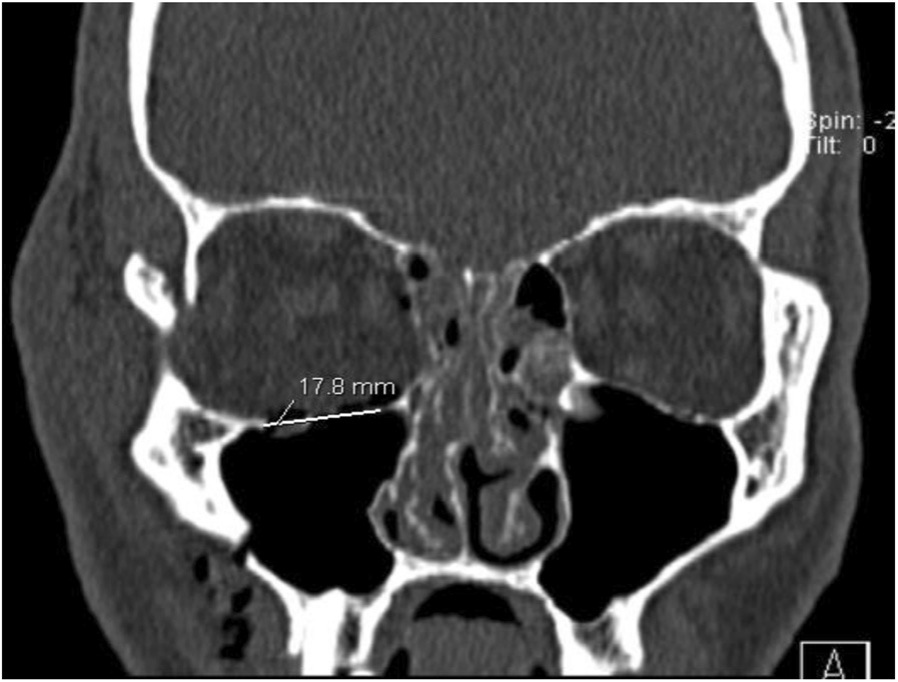
Coronal slice of a native CT-scan of the midfacial area for the measurement of orbital floor defect sizes. Shown is a zygomatic bone fracture on the right side involving the lateroorbital column, the orbital floor and the anterior maxillary sinus wall

Size of orbital floor defect was calculated analog to Ellis 3^rd^ and Tan 2003 by analyzing coronal slices of CT-scans of the orbital floor [7]. To get the correct defect size the number of slices was counted in which one could see the defect. Afterwards this number was multiplicated with the thickness of the slices and the defect size mediolateral (Fig. 1).

Additionally for patients between 2010 and 2012 with fully documented pre- and postoperative data a questionnaire of contentedness was performed (collective height: 83 patients; attendance: 63 patients) at minimum 8 month and at maximum 36 month after surgical intervention. This questionnaire aimed to get further subjective information of these patients concerning aesthetic aspects of the infraorbital pit, function (extraocular movement, diplopia, hypesthesia, lower eyelid function) and general treatment/results by using free questions, multiple choice and visual analogue scale in alteration.

### Statistics

Data analysis and presentation was performed descriptively in SPSS vers. 22 (IBM Corporation, Armonk, NY, USA) and MS Excel (Microsoft Corporation, Redmond, WA, USA) and controlled by a statistician.

## Results

### Collective

**Fig. 2:**
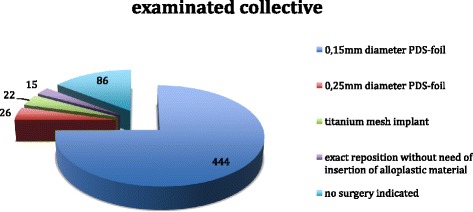
Examinated patients’ collective (*n* = 593 patients)

An over all collective of 775 patients in the years 2005 up to 2012 was examined (541 [69,8 %] male and 243 [30,2 %] female patients). Median age was 44 (mean value 46 years; minimum 3 years, maximum 94 years [standard deviation 20,87]). In 593 patients full pre- and postoperative data appropriate to inclusion criteria was available. 507 (85,5 %) of these patients underwent primary surgical treatment, 86 had no surgical indication, refused surgical treatment or had contraindications against surgery (Fig. 2). The smallest average defect one could find occurred in cases with no indication to a surgical procedure (81 mm^2^), the largest in cases with titanium mesh reconstruction (601,5 mm^2^). Time for surgical intervention ranged from average 55 min for exact reposition without need of alloplastic material insertion, 77 min using 0.15 mm diameter PDS-foil, 95 min applying titanium mesh up to 105 min inserting 0.25 mm diameter PDS-foil.

### Collective with no surgical indication

In cases with no surgical indication diplopia occurred preoperative in 10 cases with a reduction to one case 1 weeks postoperative (*p* < 0.01). Presurgical exophthalmus occurred in this collective in 2 cases, postoperative only in 1 case (not significant [n.s.]). No ex- or enophthalmus was present in any pre- and postoperative case (Table 1). Minimum defect size was 2,3 mm^2^, maximum was 515 mm^2^.

**Table 1:** Treatment depending symptoms (percentages in relation to complete collective)

Treatment	median defect size (mm^2^)	Diplopia		Exopthalmus		Enophthalmus	
		Pre-surgery	Post-surgery	Pre-surgery	Post-surgery	Pre-surgery	Post-surgery
No surgery indicated	81,0	10 (11,6 %)	1 (1,2 %)	2 (2,3 %)	1 (1,2 %)	0 (0 %)	0 (0 %)
Exact reposition without need of insertion of alloplastic material	142,8	1 (6,7 %)	0 (0 %)	0 (0 %)	0 (0 %)	0 (0 %)	0 (0 %)
0,15 mm diameter PDS-foil	267,3	74 (16 %)	21 (4,9 %)	11 (2,5)	8 (1,8 %)	4 (0,9 %)	6 (1,4 %)
0,25 mm diameter PDS-foil	437,3	9 (34,6 %)	1 (3,8 %)	1 (3,8 %)	2 (7,7 %)	1 (3,8 %)	1 (3,8 %)
Titanium mesh implant	601,5	10 (45,5 %)	7 (31,8 %)	0 (0 %)	0 (0 %)	3 (13,6 %)	0 (0 %)

### Cases with possible exact reposition and no need to insert alloplastic material

Preoperative diplopia occurred in one case, postoperatively no diplopia occurred (n.s.). No pre- or postoperative exophthalmus or enophthalmus was observed. Minimum defect size was 49 mm^2^, maximum defect size treated in this group was 450 mm^2^ (Table 1).

### Reconstruction with 0,15 mm diameter PDS-foil

Whereas 16 % (average) of the patients presented preoperative diplopia, only 4,9 % showed this postoperative which means a significant reduction (*p* < 0.01). Ex- and enophthalmus was only mentioned in a very low number of patients in this group. Consequentially no significant reduction from pre- to postoperative could be evaluated. Minimum defect size was 7,2 mm^2^, maximum defect size treated in this group was 370,8 mm^2^ (Table 1).

### Reconstruction with 0,25 mm diameter PDS-foil

In comparison to the reconstruction with the thinner diameter foil a significant reduction of diplopia pre- to postoperative (34,6 to 3,8 %; *p* < 0.01) could be obtained. Pre- and postoperative data concerning ex- and enopthalmus were corresponding to the reconstructions using the 0.15 mm PDS-foil. Minimum defect size was 100 mm^2^, maximum defect size treated in this group was 825 mm^2^ (Table 1).

### Reconstruction with titanium mesh

In comparison to reconstruction with PDS-foil a higher percentage of patients of this collective had a preoperative diplopia (45,5 %). There was no significant difference in the potential reduction compared to the postoperative stadium (31,8 %; *p* = 0,07). An enophthalmus was diagnosed preoperatively in 13,6 % of the cases presumably due to the average size of defects and was reduced in each case following surgery. Exophthalmus was no problem neither pre- nor postoperative. However the low number of patients in this group leads to limitations in validity of the data. Minimum defect size was 100.8 mm^2^, maximum defect size treated in this group was 1004 mm^2^ (Table 1).

### Patient contentedness

83 Patients in the years 2010 to 2012 additionally received a written questionnaire about postoperative symptoms and surgical contentedness. The questionnaires were handed out at a minimum of eight and at a maximum 36 months after initial trauma, to ensure documentation of potential long-term effects after injury or surgical intervention. 63 agreed in and answered this questionnaire. In 12 cases no surgical treatment was indicated, in two cases exact reposition of the fragments was possible equaling no insertion of alloplastic material. In 43 cases a 0.15 mm diameter foil was applied, two patients received a 0.25 mm diameter foil and four a titanium mesh. The impairment results are mentioned in Table 2. They mainly correlate with the short-term results. Moreover 50 % of the patients reconstructed with titanium meshes indicate foreign body sensations and cold feeling during changes in weather in the area of operation. Again it is to be mentioned that the validity of these results is limited due to the low number of patients in this collective.

**Table 2:** Intermediate-term patient impairments

Treatment	Median defect size (mm^2^)	Foreign body sensation/cold feeling when weather is changing (% of collective)	Postoperative diplopia (n)	Postoperative exophthalmos (n)	Postoperative enophthalmus (n)
No surgigal treatment indicated	135,64	0 %	0	0	0
Exact reposition without need of insertion of alloplastic material	89,9	0 %	0	0	0
0,15 mm diameter PDS-foil	319,71	4,7 %	1	1	0
0,25 mm diameter PDS-foil	448,8	0 %	0	0	0
Titanium mesh implant	481,67	50 %	0	0	0

## Discussion

Regarding the current data of this study the PDS-foil seems to be the adequate material to reconstruct orbital floor fractures with average sizes of 267 mm^2^ (0.15 mm diameter foil) up to 437 mm^2^ in average (0.25 mm diameter foil). In single cases even 0.25 mm diameter foil was able to reconstruct defects up to 825 mm^2^, probably leading to an overlapping indication in terms of patients reconstructed with titanium meshes and their average defect size of 602 mm^2^. The 0.15 mm diameter foil is able to reduce pre- to postoperative diplopia from 16 to 4,9 % along with a low tolerable rate of postoperative ex- and enophthalmus (1,8 %/1,4 %). Reconstructed patients with 0.25 mm diameter foil reveal similar results concerning pre- to postoperative reduction of diplopia. Long-term data of the patients who completed the questionnaire confirms these results even though this group was slightly smaller. Moreover reconstruction with 0.15 mm diameter PDS-foil overall requires the least time for surgical intervention being compared to reconstructions applying 0.25 mm diameter PDS-foil or titanium mesh. Our data approve the studies of Gierloff et al. and Gerressen et al. [15, 16]. However, our collective of patients with short- and long-term results is distinctively bigger especially concerning reconstructed cases using 0.15 and 0.25 mm diameter PDS foil. Thus our data enhances the long-term clinical results of Gerressen et. al. regarding reconstruction of greater defects (average 432 mm^2^ by Gerressen et al. [16], average 437 mm^2^ in our study for reconstruction with 0.25 mm diameter PDS-foil). Although our group of titanium-mesh reconstructed patients is much smaller than the one reconstructed with PDS-foil its results are remarkable: insufficient short-term reduction of diplopia and long-term foreign-body feeling. Whereas a low rate of enophthalmus which was found earlier by Rosado and de Vicente et al. was also apparent in our study, we were not able to confirm the superior reduction of diplopia for titanium meshes [9]. Our findings are contrary to a present study of Baek et al. who reported resorbable and non-resorbable implants to be equally effective concerning postoperative risks and functions [17]. Ultimately the PDS-foil seems to fit for a great range of orbital floor fractures but not for every case. One the one hand small defects perhaps do not need any implant or even a surgical intervention, which reveals our results for the groups without any intervention and exact reposition of fragments without alloplastic implant. On the other hand defects with a wide range do not reveal perhaps enough bracing for the foil and the foil for the orbital tissue lying on the foil. For these cases prefabricated meshes or even individually bent titanium meshes sometimes with required navigated insertion are necessary with reasonable postoperative symptoms [18].

Even though the number of patients in the surgical treatment groups of our study is with no doubt differing determining limited significance, our results remain remarkably and may help to establish profound treatment algorithms in future.

## Conclusions

In conclusion we suggest that reconstruction of orbital floor fractures with PDS-foil taking indication and defect size (median: 437 mm^2^ for 0.25 mm diameter PDS-foil) into account comes along with a low and acceptable rate of postoperative diplopia, ex- and enophthalmus in the majority of cases. Particularly the 0.15 mm diameter PDS-foil seems to generate the best clinical results including median orbital floor defects with a size of 250 to 300 mm^2^ resulting in the lowest postoperative rate of diplopia and en- and/or exophthalmus.

The indication for a reconstruction with titanium meshes (even cases with individually bent meshes with or without navigated insertion) should be reduced to cases of extensive fractures keeping the insufficient reduction of diplopia and the remarkably high percentage of foreign body sensations in mind.

## Ethics

This retrospective study has been approved by the ethics committee of the medical faculty of the university of Duesseldorf (study number 4222, year 2013).
